# Immunosuppressant activity and morphological changes in *Leishmania amazonensis* treated with extracts from seeds of *Lonchocarpus cultratus*

**DOI:** 10.22038/ajp.2025.26025

**Published:** 2026

**Authors:** Fernanda Weyand Banhuk, Izabela Virginia Staffen, Fernanda Tomiotto-Pellissier, Bruna Taciane da Silva Bortoleti, Wander Rogério Pavanelli, Thaís Soprani Ayala, Rafael Andrade Menolli

**Affiliations:** 1 *Laboratory of Applied Immunology, Center of Medical and Pharmaceutical Sciences, State University of West Paraná, Cascavel, Paraná, Brazil*; 2 *Biosciences and Biotechnology Postgraduate Program, Carlos Chagas Institute, Curitiba, Paraná, Brazil*

**Keywords:** Lonchocarpus, Leishmaniasis, Seed extracts, Lonchocarpine, Cytotoxicity, Leishmanicidal

## Abstract

**Objective:**

The first-line drugs used for treating leishmaniasis are highly costly and aggressive. Extracts from *Lonchocarpus cultratus *have trypanocidal activity and possess several compounds with biological activities. This study sought to observe the *in vitro* anti-*Leishmania amazonensis* action of extracts from seeds of *L. cultratus*. Furthermore, the immunomodulatory and antioxidant characteristics of the extracts were determined.

**Materials and Methods:**

Sequential extraction with hexane, dichloromethane, and methanol was performed to obtain extracts from *L. cultratus *seeds, which were characterized via ^1^H NMR. Promastigotes, intracellular amastigotes, and murine macrophages were treated with increasing concentrations of the extracts, and the inhibition rates were determined by scanning electron microscopy (SEM) analysis of the extracellular forms of the extracts. The immunomodulatory activity of the extract was determined against stimulated RAW macrophages.

**Results:**

Isocordoin and lonchocarpine were identified in dichloromethane and hexane extracts. Dichloromethane (LDS), hexane (LHS), and methanolic (LMS) extracts inhibited promastigote cell growth (IC_50_ values of 5.18±1.18, 5.25±1.47, and 33.89±1.62 μg/ml, respectively) and decreased the number of amastigotes in the macrophages (IC_50_ values of 1.41±0.31, 6.33±1.42, and 5.87±1.37 μg/ml, respectively). Hexane and methanolic extracts showed low toxicity in macrophages, resulting in a high selectivity index against promastigotes and amastigotes. In addition, the three extracts immunomodulated macrophages, reducing nitric oxide (NO) secretion.

**Conclusion:**

The results revealed that the activities of the *L. cultratus *extracts included leishmanicidal effects, low cytotoxicity to macrophages, and immunosuppression *in vitro*.

## Introduction

Leishmaniasis is a neglected tropical disease (NTD) and is considered a significant public health problem that comprises a spectrum of infections caused by protozoa of the *Leishmania* genus (Anversa et al. 2018). Promastigote forms are transmitted to the vertebrate host by the bite of a female phlebotomine sandfly (Santin et al. 2009), and once inside the host, they differentiate into amastigotes. These amastigotes are the main targets of chemotherapy for leishmaniasis (Cunningham 2002).

Few drugs are available for treating cutaneous leishmaniasis, and these drugs present toxicity, low efficacy, and difficulty in administration (Rath et al. 2003; Alvar et al. 2006). Therefore, identifying alternative treatments that are less toxic and have leishmanicidal activity similar to that of conventional treatments is mandatory.

*Lonchocarpus cultratus* (Fabaceae) is a plant found predominantly in South America (Silva and Tozzi 2012). In Brazil, it is known as “*embira*” or “*falso* *timbó*”. Several compounds have been identified in this species, and some of their biological activities have been characterized. Several biological activities have already been identified in plants belonging to the *Lonchocarpus* genus (Cassidy and Setzer 2012). More specifically, Mello et al. (1974) reported that several chalcones can be extracted from *L. cultratus* (Méllo et al. 1974). Furthermore, some studies have detected antiprotozoal activity, justifying the search for an anti-*Leishmania* compound in this species, since chalcones are frequently found in the genus *Lonchocarpus*, a metabolite with leishmanicidal effects already reported in the literature (Borges-Argáez et al. 2009).

Nuclear magnetic resonance (NMR) spectroscopy is the most commonly used approach to identify low-molecular-mass compounds, as ^1^H is highly abundant in nature; one spectrum can be acquired in a few minutes (Ocampos et al. 2024). NMR is a highly reproducible technique that is perfectly appropriate for the identification of natural extracts, as complex mixtures are often prepared via extraction to concentrate the components thought to be responsible for the desired benefits (Pauli et al. 2005). Thus, this technique is capable of identifying and quantifying components in complex mixtures such as plant extracts, providing identity, purity, strength, and composition (Colson et al. 2015).

Therefore, this study aimed to investigate the leishmanicidal, immunomodulatory, and cytotoxic activities of three extracts from *L. cultratus* seeds characterized via the NMR technique.

## Materials and Methods

### Vegetable samples

Seeds from an *L. cultratus* sample were collected from a park in Cascavel-PR, a city in southern Brazil (S 24.96308° and O 53.43674°), in May 2018. The voucher specimen was deposited in the Unioeste Herbarium (UNOP No. 20). Access to the botanical material was authorized (registration number A903B10) by Conselho de Gestão do Patrimônio Genético (CGEN/SISGEN).

### Plant extract collection and characterization

The seeds were subjected to successive extractions with hexane, dichloromethane, and methanol as the solvents, and three extracts, LHS, LDS, and LMS, respectively were obtained.

NMR spectra were obtained via Varian (Mercury Plus, BB 300 MHz) and Bruker (500 MHz) spectrometers at the State University of Maringá-PR (UEM), Brazil, using CDCl_3_ as the solvent and trimethylsilane (TMS) as an internal reference.

The chemical characterization of each extract (LHS, LDS, and LMS) was performed via ^1^H NMR, and the signals were compared with those described in the literature.

### Anti-Leishmania (L) amazonensis activity

#### Parasite maintenance

Promastigote forms of *L. amazonensis* (MHOM/BR/1977/LTB0016) were maintained with weekly subcultures in Roswell Park Memorial Institute (RPMI) culture media supplemented with 10 % of Fetal Bovine Serum (FBS) at 25°C. Promastigote experiments used the parasite at the log growth stage (3-day culture), and amastigote experiments were used in the stationary growth phase (6-day culture).

### Anti-promastigote assay

*L. amazonensis* promastigotes (1×10^5^ cells/well) were treated with *L. cultratus* extracts at concentrations ranging from 1–175 μg/ml. Parasites were counted after 24 to 96 hr of treatment. RPMI medium was used as the control, dimethyl sulfoxide (DMSO) (0.6%) in the medium was used as the vehicle control, and amphotericin B (AMP, a reference drug for leishmaniasis (Cristalia Pharmaceuticals), 0.05–10 μg/ml) was used as the positive control. Three experiments were performed in triplicate for each extract.

The IC_50_ (half maximal inhibitory concentration) was determined for the promastigotes on the basis of the mean percentage reduction in the parasite concentration compared with that of the untreated control. The data were evaluated via nonlinear regression in GraphPad Prism 8.0.

### Morphological analysis of promastigotes by scanning electron microscopy

The morphology of the promastigote forms was analyzed according to Tomiotto-Pellissier et al. (2018). Briefly, promastigotes (1×10^6 ^cells/ml) were incubated with 5 μg/ml LDS, 5 μg/ml LHS or 30 μg/ml LMS (near the IC_50_) at 25°C for 24 hr. The cells were assembled, placed in poly-L-lysine-treated coverslips, and observed by scanning electron microscopy (SEM) (FEI QUANTA 200). At least three pictures of each condition were subjected to measurement.

### Animals

Female BALB/c mice (6–8 weeks of age) were kept at 23±2°C under a 12 hr light/dark cycle and were given water and commercial feed *ad libitum*. This study was approved by the Animal Use Ethics Committee of Unioeste, Brazil (CEUA), process number 29/19, authorized on August 29, 2019.

### Antiamastigote assay

The antiamastigote assay was performed as described by Staffen et al. (2022) . Peritoneal macrophages were infected with *L. amazonensis* promastigotes (1 × 10^6^ cells/well) for six hours at 34°C and 5% CO_2_. After infection, the following treatments were added to the cells: LHS and LMS (1, 10, 15, 50, 100, 150, or 175 μg/ml), LDS (1, 10, or 15 μg/ml), RPMI 1640 medium (control), RPMI 1640 medium plus DMSO (0.6% - vehicle control) or Glucantime (GLU) (Sanofi Aventis) at the same concentrations as the extracts (positive control) for 48 hr. Subsequently, the cells were stained with Giemsa, and at least 200 cells were counted through an optical microscope (Olympus Model CBA). Two experiments were performed in triplicate for each extract.

The leishmanicidal effects of the extracts were determined based on the number of amastigotes in 100 macrophages. The IC_50_ was calculated for the amastigote forms based on the mean percentage reduction in the parasite concentration compared with that of the untreated control. The data were evaluated via nonlinear regression in GraphPad Prism 8.0.

### Viability of peritoneal macrophages

The effects of *L. cultratus* extracts on cell viability were tested in peritoneal macrophages via the 3-(4,5-dimethylthiazol-2-yl)-2,5-diphenyltetrazolium bromide (MTT) assay (Mosmann 1983). The macrophages were treated with each extract, GLU, or AMP. Untreated cells were used as controls, and RPMI 1640 plus DMSO (0.6%) was used as the vehicle control. Three experiments were performed in triplicate for each extract.

The cytotoxicity of the extracts is presented as the concentration able to kill 50% of the macrophages (CC_50_) after 48 hr. The CC_50_ was calculated via nonlinear regression in GraphPad Prism 8.0. The selectivity index (SI) was determined by the relationship between the CC_50 _and the leishmanicidal activity (IC_50_) (SI=CC_50_/IC_50_).

### Measurement of nitric oxide (NO) levels

Peritoneal macrophages were extracted as described previously. The cells were subsequently incubated for 48 hr in the following treatments: LDS, LHS, or LMS; lipopolysaccharide (LPS) (0.1 g/ml), RPMI 1640 medium, or RPMI medium plus DMSO (0.6% - vehicle control). NO secretion was determined in the cell supernatants via the Griess reaction (Green et al. 1982). The results are expressed as μmol in 2 × 10^5^ cells. Three experiments were performed in triplicate for each extract.

### Determination of the immunosuppressant activity of the extracts

The capacity of the extracts to interfere with NO secretion was verified in RAW 264.7 macrophages. A total of 5×10^5^ cells/well were plated in a 24-well plate. After 12 hr of adherence, the cells were treated with RPMI medium only as the negative control, with RPMI and LPS (100 ng/mL) as the stimulus control, with RPMI plus DMSO (0.6%) and LPS (100 ng/ml) as the vehicle control, and with different concentrations of the test compounds (LDS, LHS, and LMS) simultaneously with LPS (0.1 g/ml). The concentrations of each extract used in these experiments were selected based on the cytotoxicity results. As previously described, the nitrite concentration was determined after 48 hr. Three experiments were performed in triplicate for each extract.

### Measurement of antioxidant activity

The free radical DPPH (2,2-diphenyl-1-picrylhydrazyl) was used to measure the scavenging activity as described by Arasu et al. (2014) (Arasu et al. 2014), with modifications. The treatments were added to the following tubes: extracts (5-175 μg/ml), water (negative control), water plus DMSO (0.6%-vehicle control) or butylated hydroxytoluene (BHT) at the same concentrations as the extracts (positive control), and DPPH was added to all the tubes; after 30 min in the dark, the samples were read at 517 nm. The scavenging ability was calculated via the following equation:

=(1-((abs sample-blank)/(abs negative control))) ×100

Three experiments were performed in triplicate for each extract, and the scavenging activity achieved at each different concentration was used to calculate the EC_50_ values via nonlinear regression in GraphPad Prism 8.0.

### Statistical analysis

The normality of the data was tested via the Shapiro‒Wilk test. Parametric data were analyzed with ANOVA with Dunnett's multiple comparisons *post hoc* test. Nonparametric results (promastigotes- Figure 2, amastigotes-Figure 4, and inhibition test (LDS and LHS-Figure 5) data) were analyzed with the Kruskal‒Wallis test with Dunn's post hoc test. GraphPad Prism 8.0 was used for analysis, and a significance level of 95% (p value≤0.05) was adopted.

## Results

### Different phytochemical compositions of LDS, LHS, and LMS

*L. cultratus* seeds provided 95.94 g of LHS, resulting in a yield of 30.63%, while from LDS and LMS were obtained 8.17 g and 16.45 g, with yields of 2.61% and 5.25%, respectively.

**Supplementary Figure1:**
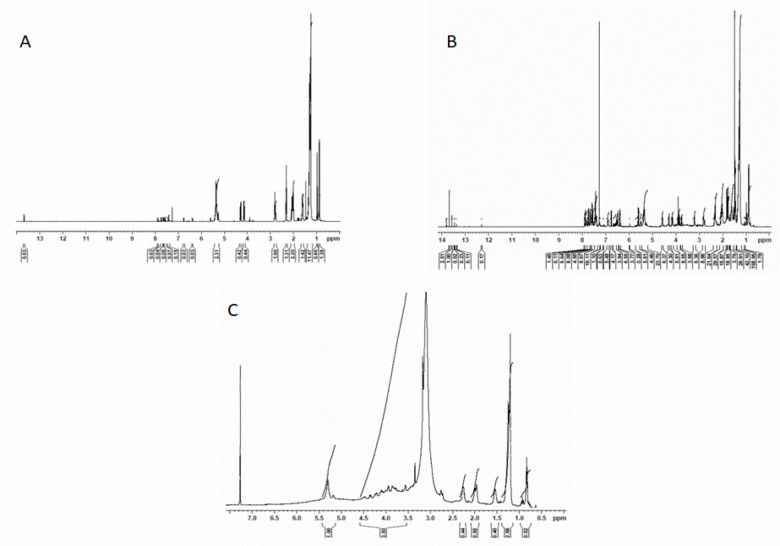
^1^H nuclear magnetic resonance (NMR; 500 MHz) spectra of the LHS, LDS, and LMS extracts from the seeds of *L. cultratus*. A: hexane extract (LHS) at CD_3_OD as the solvent. B: dichloromethane extract (LDS) at CDCl_3_ as the solvent. C: methanolic extract (LMS) at CDCl_3_ as the solvent. Chemical shifts are expressed in  , ppm.

The ^1^H NMR (500 MHz) spectra of the hexane, dichloromethane, and methanolic extracts revealed that the LHS and LDS contain primarily low- and medium-polarity substances such as those extracted by apolar solvents such as hexane and dichloromethane, which were used to obtain LHS and LDS (Supplementary Figure 1).

**Supplementary Figure 2:**
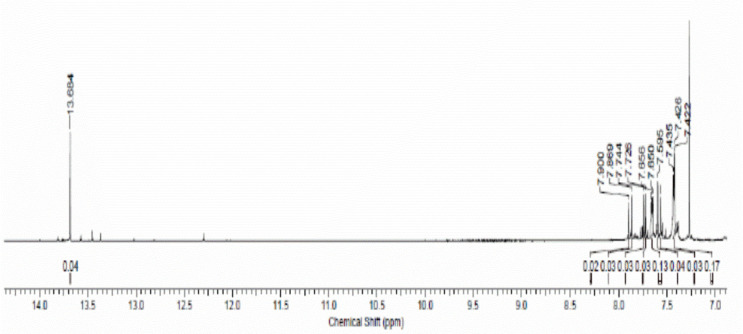
^1^H nuclear magnetic resonance (NMR; 500 MHz) spectrum (high field) of the LHS extract from the seeds of *L. cultratus*. CD_3_OD was solvent. Chemical shifts are expressed in  , ppm.

**Supplementary Figure 3:**
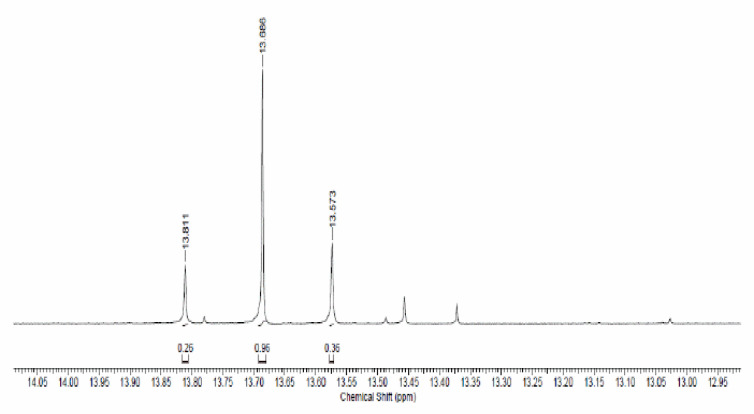
^1^H nuclear magnetic resonance (NMR; 500 MHz) spectrum (high field) of the LDS extract from the seeds of *L. cultratus*. CD_3_OD was solvent. Chemical shifts are expressed in  , ppm.

The LHS and LDS spectra showed a signal at 13.68 ppm (Supplementary Figures 2 and 3), indicating that the hydrogens were strongly related to encounters, such as the hydroxyl between hydrogen and C=O. The peaks observed in the ^1^H NMR spectra of the dichloromethane and hexane extracts indicate the presence of lonchocarpine according to a comparison of the data with those described in the literature (Borges-Argáez et al. 2002; Lima et al. 2013) (Table 1). Additionally, compared with those in the literature, the signals obtained from the LDS extract spectrum revealed the presence of isocordoin (Caamal-Fuentes et al. 2015; da Silva Landim et al. 2021) (Table 2).

 The LMS ^1^H NMR spectrum was different from the other two extracts spectra, with the absence of peaks in the high-field region, indicating the absence of chalcones. LMS signals appear between 5.50 and 0.80 ppm (Supplementary Figure 1) and indicate a polyhydroxy alkaloid (pyrrolidine type). These compounds possess hydroxyl groups that generally appear at low frequencies (3.5–4.5 ppm) in the molecular spectrum. The remaining protons appear in the region of 1–3 ppm as overlapping signals at low field strengths (Pant et al. 2000). Several peaks between 2.7 and 4.0 ppm are consistent with the CH-OH protons of hydroxy pyrrolidine alkaloids, as shown by Welter et al. (1976).

**Table 1:** ^1^H NMR spectra of lonchocarpine and the dichloromethane (LDS) and hexane (LHS) extracts from L. cultratus

	Lonchocarpine*	**LDS**	**LHS**
	**δ ** ^1^ **H (** ***J*** ** Hz)**	**δ ** ^1^ **H (** ***J*** ** Hz)**	**δ ** ^1^ **H (** ***J*** ** Hz)**
1	-	-	-
2,6	7,62 (m)	7,65 (m)	7,65 (m)
3,5	7,40 (m)	7,40 (m)	7,42
4	7,40 (m)	7,40 (m)	7,42
α	7,54 (d, 15,5)	7,57 (d, *J*)	7,57 (d, *J*)
β	7,85 (d, 15,5)	7,88 (d, *J*)	7,88 (d, *J*)
C=O	-	-	-
1’	-	-	-
2’	-	-	-
3’	-	-	-
4’	-	-	-
5’	6,37 (d, 8,9)	6,39 (d, *J*)	6,39 (d, *J*)
6’	7,70 (d, 8,9)	7,74 (d, *J*)	7,73 (d, *J*)
1”	-	-	-
2”	-	-	-
3”	5,57 (d, 10)	5,61 (d, *J*)	5,60 (d, *J*)
4”	6,74 (d, 10)	6,76 (d, *J*)	6,76 (d, *J*)
5”	1,45 (s)	1,48	1,47
6”	1,45 (s)	1,48	1,47
OCH_3_	-	-	-
2’-OH	13,67 (s)	13,68 (s)	13,68 (s)

*The data for lonchocarpine were acquired from Borges-Argáez et al., 2002 (11) and Lima et al., 2013(12) (500 MHz/CDCl_3_); LDS and LHS (500 MHz/CDCl_3_).

**Table 2:** ^1^H NMR spectra of isocordoin and the compound present in dichloromethane (LDS) extract of L. cultratus.

	Isocordoin*	**LDS**
	δ ^1^H (*J* Hz)	δ ^1^H (*J* Hz)
1	-	-
2,6	7,43 m	7,44
3,5	7,43 m	7,43
4	7,43 m	7,43
α	7,60 d (15,4)	7,60 d
β	7,89 d (15,4)	7,90 d
C=O	-	-
1’	-	-
2’	-	-
3’	-	-
4’	-	-
5’	6,44 d (8.9)	6,39 d
6’	7,74 d (8,9)	7,75 d
1”	3,49 d (7,0)	3,51 d
2”	5,31 t (7,0)	5,34 t
3”	-	-
4”	1,85 s	1,85 s
5”	1,78 s	1,78 s
6”	-	-
OCH_3_	-	-
2’-OH	13,76 s	13,81 s

*Isocordoin: Borges-Argaez et al. 2002 (11) (CDCl3, 500 MHz). LDS (500 MHz/CDCl_3_): dichloromethane extract.

### Effects of the L. cultratus extracts on the viability of peritoneal macrophages

**Figure 1:**
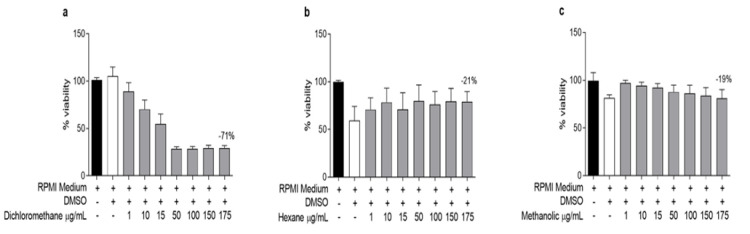
Effects of (a) dichloromethane (LDS), (b) hexane (LHS), and (c) methanolic (LMS) extracts from L. cultratus seeds on the viability of murine macrophages. The results are expressed as the mean ± SEM of three independent experiments performed in triplicate. The controls were RPMI medium or medium supplemented with DMSO (0.6%). *, *** and **** p<0.05, p<0.001, and p<0,0001, respectively, compared with the negative control (199 medium).

Table 3 shows the cytotoxic concentrations of the extracts for 50% of the macrophages (CC_50_). The LHS and LMS extracts did not demonstrate any toxicity to the macrophages (Figure 1 b-c), as they maintained cell viability even at the highest concentration (175 µg/ml). LDS at concentrations of 50, 100, 150, and 175 µg/ml was toxic to the cells (Figure 1a).

The CC_50 _values indicated that LDS had the highest toxicity, reaching 9.08 µg/ml, while the other two extracts had toxicity at levels >300 µg/ml. Although the highest value used to determine the CC_50 _of the extracts was 175 µg/mL, the minimal toxicity demonstrated by LHS and LMS at this concentration led to the conclusion that the CC_50 _was at least 300 µg/ml.

The drugs used as treatment controls (GLU and AMP) were tested against macrophages, and GLU had no toxic effects on the cells (data not shown), with a CC_50_ above 300 µg/ml (Table 3); however, the cytotoxicity of AMP was confirmed (CC_50_ of 0.097 µg/ml) (Table 3).

**Table 3:** IC_50_ and CC_50_ values reached by the dichloromethane (LDS), hexane (LHS), and methanolic (LMS) extracts from L. cultratus seeds on the promastigote and amastigote forms of L. amazonensis and murine macrophages and the respective selectivity index (SI).

**Extracts**	**Promastigotes (96 hr)** **IC**_50 _ **(µg/ml)**	**Amastigotes (48 hr)** **IC**_50 _ **(µg/ml)**	**Macrophages BALB/c** **CC**_50 _ **(µg/ml)**		**SI **_(promastigotes)_	**SI** _(amastigotes)_
LDS	5.18±1.18	1.41±0.31	12.8±1.44	2.47		9.08
LHS	5.25±1.47	6.33±1.42	>300	>57.14		>43.29
LMS	33.89±1.62	5.87±1.37	>300	>8.85		>51.11
AMP	0.052±0.02	-	0.097±0.02	1.82		-
GLU	-	12.18±1.62	>300	-		>24.63

AMP – Amphotericin, and GLU – Glucantime

### L. cultratus extracts inhibit the proliferation of L. amazonensis promastigotes and act on intracellular amastigotes

**Figure 2:**
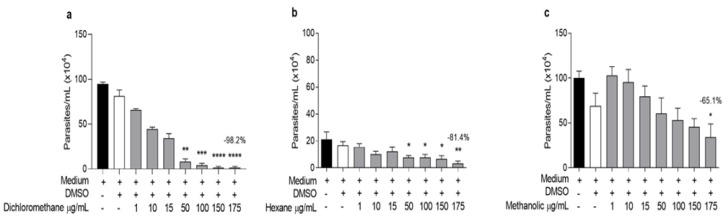
Effects of (a) LDS (dichloromethane), (b) LHS (hexane), and (c) LMS (methanolic) extracts on L. amazonensis promastigotes. The results are expressed as the mean ± SEM of three independent experiments performed in triplicate. *, **, *** and **** p<0.05, p<0.01, p<0.001, and p<0,000, respectively, compared with the negative control (199 medium).

**Supplementary Figure 5:**
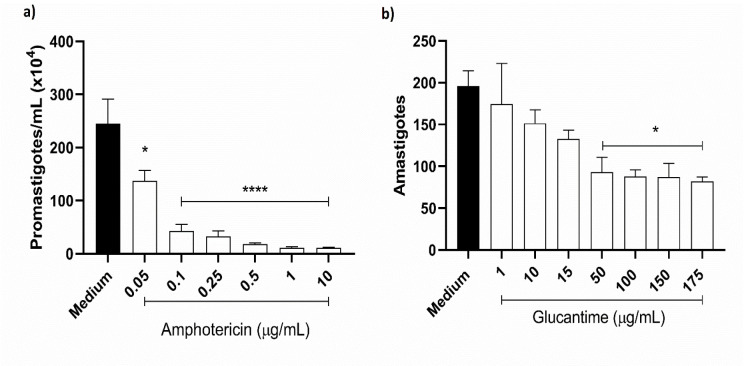
The anti-*L. amazonensis* activity of amphotericin on promastigotes (a) and Glucantime® on amastigotes (b) forms after 96h (a) or 48h (b) of treatment. The values are mean ± SEM of three independent experiments performed in triplicate. *Significantly different from the Medium, *p <0.05 and **** *p* <0.0001. Medium: RPMI.

*L. cultratus* extracts promoted growth inhibition over promastigote forms of 65.9, 98.2, and 81.4% after 96 hr (LMS, LDS, and LHS, respectively) at the relatively high concentration (175 µg/ml) (Figure 2 a-c), indicating that LDS almost completely inhibited the parasite, similar to the positive control AMP (95.5%) (Supplementary Figure 5) at the highest concentration (10 µg/ml).

The inhibitory activity of LDS and LHS started at 50 µg/ml, and LMS at 175 µg/ml significantly inhibited the growth of the promastigotes (p<0.05) (Figure 2 a-c) compared with that of the control (untreated) group, whereas the DMSO group did not, demonstrating that the leishmanicidal activity of the extracts was not altered by the solvent. 

The IC_50 _values, calculated from the inhibition percentages, are shown in Table 3. The LDS and LHS extracts had IC_50_ values close to those calculated for the promastigote form (5.18±1.18 and 5.25±0.78 µg/ml, respectively), which were greater than those of the AMP (0.052±0.02 µg/ml), a potent leishmanicidal agent *in vitro*. Compared with the other two extracts, the LMS extract had a lower inhibitory effect.

**Figure 3:**
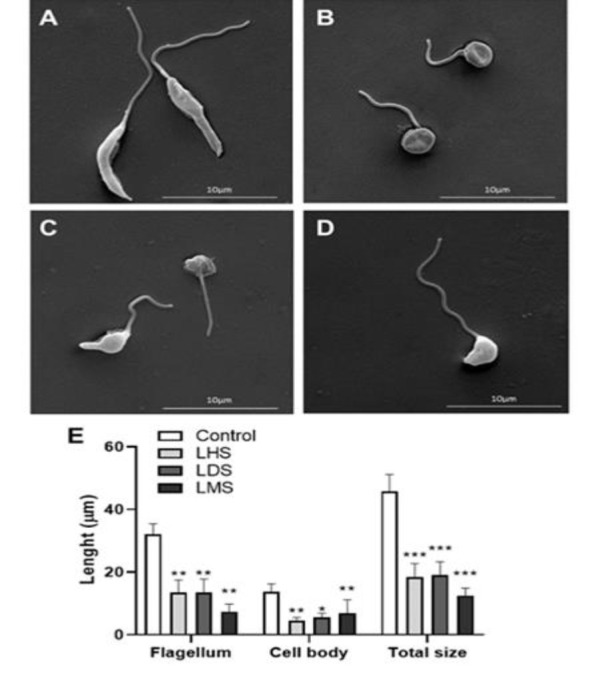
SEM (scanning electron microscopy) pictures exhibiting changes in L. amazonensis promastigotes after 24 hr of incubation with 5 μg/ml LHS or LDS, or 30 μg/ml LMS. A) Promastigotes in the absence of extracts (Medium - Control); B–D) Promastigotes incubated with LHS (B), LMS (C), or LDS (D); E) Body and flagellum extent of promastigotes incubated with or without LHS, LDS, or LMS; *Different from the Control, *p<0.05, **p<0.01, and ***p<0.001.

The morphology and size of the parasites were evaluated via SEM, and the images revealed that the untreated parasites had a characteristic morphology, with a long cell body and undamaged plasma membrane. The cells treated with the extracts presented changes in their form. These compounds led to roundness of the protozoan cells and abnormalities in the plasma membrane (Figure 3A-D), besides to significant decreases in body length (p<0.05) and total length (p<0.001). Additionally, the flagella of the parasites treated with the three extracts substantially decreased in size (p<0.01) (Figure 3E).

**Figure 4:**
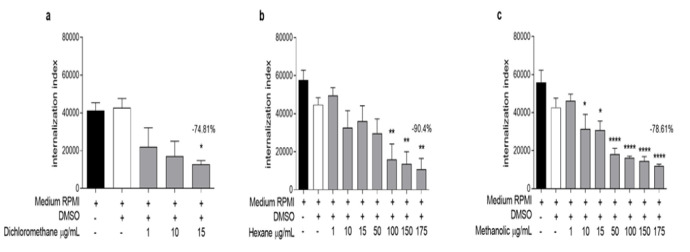
Effects of (a) dichloromethane (LDS), (b) hexane (LHS), and (c) methanolic (LMS) extracts from L. cultratus seeds on the internalization index (number of infected macrophages × number of amastigotes) of L. amazonensis. The results are expressed as the mean ± SEM of two experiments. *Different from the control (medium RPMI), *p<0.05, **p<0.01, and **p<0.001.

The extracts were also evaluated against intracellular forms of *L. amazonensis,* and all of them showed significant differences in the internalization indices (number of infected macrophages × the number of amastigotes) (Figure 4 a-c). LDS was more active against amastigotes, with an IC_50_ of 1.41±0.31 µg/ml (Table 3) and 74.81% inhibition at 15 µg/ml. LHS at 100, 150, and 175 µg/ml had significant differences in the internalization index compared to control, with IC_50_ values of 6.33±1.42 and 90.4% inhibition of amastigotes at 175 µg/ml. LMS significantly differed from the negative control starting at 10 µg/ml, with an inhibition rate of 78.61% at the highest concentration and an IC_50_ of 5.87±1.37. The three extracts had better effects than did the positive control in the amastigote experiments (Supplementary Figure 5), which was unexpected because GLU can activate macrophages but not the extracts used (Mookerjee Basu et al. 2006; Griebler et al. 2021). 

### Extracts from L. cultratus seeds inhibited NO secretion from LPS-stimulated RAW 264.7 macrophages

**Figure 5:**
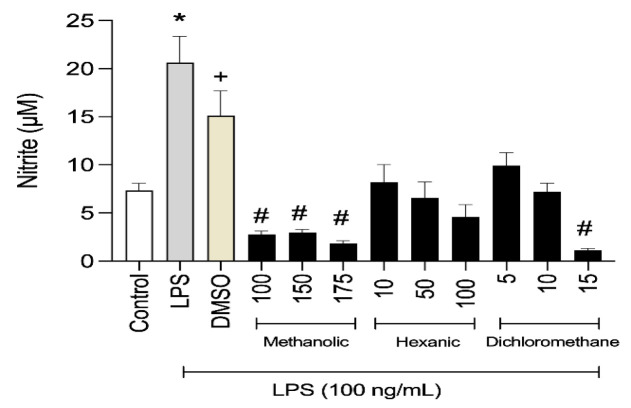
Effects of (a) dichloromethane (LDS), (b) hexane (LHS), and (c) methanolic (LMS) extracts (µg/ml) from L. cultratus seeds on NO secretion from RAW 264.7 cells under LPS stimulation for 48 hr. The results are expressed as the mean ± SEM of three independent experiments performed in triplicate. * Indicates a significant difference (p<0.05) compared with the control and extracts; ^+ ^indicates no significant difference (p>0.05) in response to LPS. ^# ^indicates a significant difference (p<0.05) compared with the DMSO control.

In response to the immunosuppressive action of the extracts, RAW264.7 cells stimulated with LPS and simultaneously treated with LHS, LDS, and LMS, secreted significantly less NO after 48 hr (Figure 5). The NO production of the cells treated with the DMSO control (0.6% DMSO and LPS) did not differ from that of the positive control, demonstrating that the diluent did not interfere with LPS stimulation.

### Antioxidant activity

**Figure 6:**
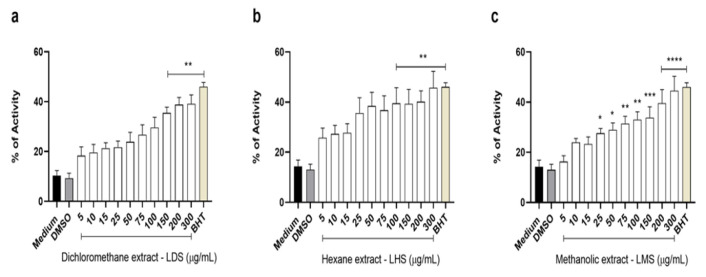
Percentages (±SEM) of antioxidant activity of the LDS (A), LHS (B), and LMS (C) extracts. Butylated hydroxytoluene (BHT) (positive control, 5 µg/ml) was used. *Different from the negative control (medium). *p<0.05, **p<0.01, ***p<0.001, and ****p<0.0001.

The antioxidant ability of the *L. cultratus* extracts was tested via the DPPH test (Figure 6). The hexane extract (LHS) showed higher activities; the greater concentrations of LDS, LHS, and LMS had scavenging abilities of 39.06, 45.74, and 44.53%, respectively. The positive control, BHT, has a very strong antioxidant capacity and had 46.02% activity at 5 µg/ml (Figure 6), whereas at 300 µg/mL, it had 92.86% activity (data not shown). The EC_50 _was calculated for three extracts, and LHS had the highest EC_50_, reaching 43.44±1.23 for LMS, 31.47±1.24 for LDS, and 14.98±1.42 for LHS, whereas BHT had an EC_50 _of 6.14±1.05 µg/ml.

## Discussion

The presence of chalcones in *Lonchocarpus *species is expected, as shown by an extensive revision of the genus by Cassidy and Setzer (2012), which revealed isocordoin in *L. floribundus*, *L. neuroscapha*, *L. sericeus*, and *L. xuul* and lonchocarpine in *L. floribundus*, *L. montanus,* *L. neuroscapha*, *L. sericeus*, and *L. xuul*.

The substantial toxicity presented by LDS can be explained by the presence of isocordoin, as this compound which was isolated from the hexane extract of *L. xuul*, demonstrated toxicity *in vitro* (Borges-Argáez et al. 2009). Furthermore, the other two compounds in LDS (derricin and lonchocarpine) were not toxic to mammalian tumor cells when obtained from *L. sericeus*. The presence of lonchocarpine in LHS did not cause toxicity, in agreement with other studies, in which the presence of this compound also did not alter cell viability (Jeong et al. 2017).

Chalcones naturally produced by plants, such as isocordoin and lonchocarpine, do not show intense anti-*Leishmania *activity when evaluated alone. Indeed, the extracts from *L. cultratus *seeds contain a mixture of substances including chalcones in LDS and LHS, demonstrating a synergistic effect between the compounds present. Furthermore, Osorio et al. (2007) classified the extracts as very active when the IC_50 _values against promastigote forms were <10 µg/ml and as active when 50 > IC_50 _> 10 µg/ml, which indicates that LHS and LDS have substantial activity against *L. amazonensis *promastigotes, whereas LMS is less effective in this situation.

The form and dimension, flagellum extent, and ultrastructural features define *Leishmania* parasite cell morphology (Sunter and Gull 2017). Therefore, the changes observed via electron microscopy indicate that the extracts affect the extracellular structures of the parasites. Therefore, in this study, the parasite was subjected to conditions in which the metacyclic form (more infectious) was obtained on the fifth day of culture (Sunter and Gull 2017).

An antileishmanial drug should hit the intracellular form of *L. amazonensis* in *in vitro* experiments to be a candidate for an *in vivo* trial; furthermore, natural extracts against amastigote forms have been evaluated (Maleki et al. 2024). To characterize a good candidate, some parameters *in vitro* must be met, demonstrating an established leishmanicidal action on the amastigote forms (IC_50_ < 3 µg/ml and SI > 10) (Ioset JR, Brun R, Wenzler T, Kaiser M 2009). The dichloromethane and hexane extracts from *L. cultratus* reached values close to these conditions (IC_50_ LDS 5.18 and LHS 5.25 µg/ml; SI LDS 9.08 and LHS>43.29) and are candidates for experimental *in vivo* studies. The methanolic extract did not achieve the desired IC_50_; however, its SI was much greater (>51.11) than requested (Passalacqua et al. 2015).

NO plays the main role in eliminating intracellular parasites via the immune response which involves phagocytosis through macrophages. In addition, these cells can contribute to the response by secreting diverse mediators which can contribute to leishmanicidal action (Tomiotto-Pellissier et al. 2018). The immunomodulatory capacity of chalcones has already been proven, as various derivatives have been shown to affect macrophages (Mohd Fauzi et al. 2015). This immunosuppressive effect was demonstrated by Park et al. (2009) who reported that a reduction in iNOS expression occurred through a block in Activator Protein 1 (AP-1), a transcription factor that stimulates iNOS. Another chalcone (lonchocarpine) was able to decrease the secretion of NO, Tumor Necrosis Factor alpha (TNF-α), and Interleukin 6 (IL-6) in microglia in a dose-dependent manner, suppressing NF-κB signaling (Jeong et al. 2017). Therefore, the immunosuppressive ability observed in the LDS and LHS groups, which inhibited NO secretion in the macrophages stimulated with LPS, may be attributed to isocordoin and lonchocarpine, the chalcones detected in the extracts. Furthermore, LMS suppressed NO secretion, and although it does not contain chalcones, it contains polyhydroxy pyrrolidine alkaloids which have anti-inflammatory characteristics (Wu et al. 2011). Thus, immunomodulatory drugs should be used together as antiparasitics because modulation of the immune response may prevent lesions caused by the immune system but, on the other hand, permit pathogen growth, contributing to the permanence of the parasites. 

Polyphenols and alkaloids stand out as natural products with intense antioxidant activity. These two groups are secondary metabolites present in many plants (Efiom Okokon et al. 2013). The antioxidant capacity of these groups is based on various mechanisms, such as coupling to transition metals, scavenging free radicals, and inhibiting enzymes (Chang et al. 2011). Oxidative stress actively participates in the immune response, contributing to the elimination of microorganisms and causing the release of free radicals during this process. Thus, natural substances with antioxidant activity *in vitro* can be considered candidates for compounds with anti-inflammatory activity *in vivo*, as observed here with *L. cultratus.*

Dichloromethane, hexane, and methanolic extracts from *L. cultratus *seeds demonstrated leishmanicidal effects on the infective parasite, with the methanolic fraction showing low cytotoxicity. The LDS and LHS extracts had significant effects on the promastigote form, but SEM demonstrated that the size and morphology of the parasites were altered by the three extraction methods. In addition, these compounds exhibited immunosuppressant activity *in vitro*, inhibited NO secretion from macrophages stimulated by LPS, and exhibited antioxidant activity.

The literature shows that the compounds identified in LDS, LHS, and LMS have weak leishmanicidal activity. However, the synergy between the compounds was clear in terms of their anti-*Leishmania* activity. Thus, the interactions of these compounds should be addressed by evaluating their ability to inhibit protozoans alone or in combination, confirming their role.

## Figures and Tables

**Supplementary Figure 4:**
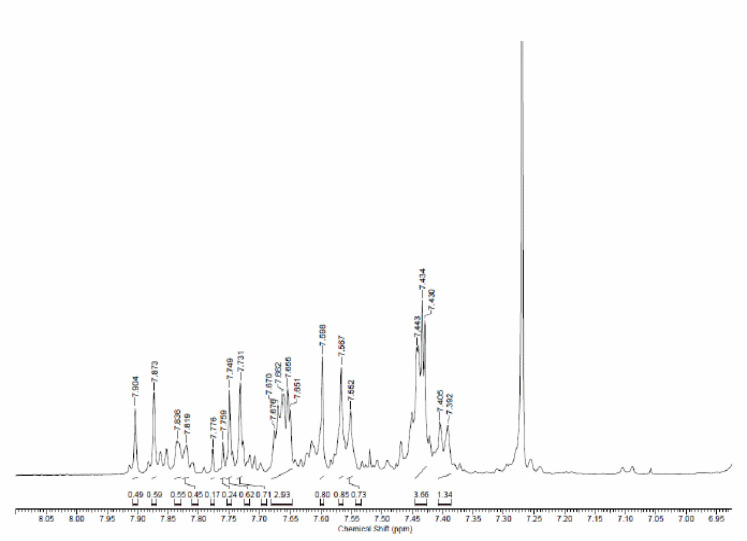
^1^H nuclear magnetic resonance (NMR; 500 MHz) spectrum (expansion) of the LDS extract from the seeds of *L. cultratus*. CD_3_OD was solvent. Chemical shifts are expressed in  , ppm.
